# Position and Orientation Tracking in a Ubiquitous Monitoring System for Parkinson Disease Patients With Freezing of Gait Symptom

**DOI:** 10.2196/mhealth.2539

**Published:** 2013-07-15

**Authors:** Boris Takač, Andreu Català, Daniel Rodríguez Martín, Nico van der Aa, Wei Chen, Matthias Rauterberg

**Affiliations:** ^1^Technical Research Centre for Dependency Care and Autonomous LivingUniversitat Politècnica de Catalunya - BarcelonaTechVilanova i la GeltrúSpain; ^2^Department of Industrial DesignEindhoven University of TechnologyEindhovenNetherlands; ^3^Noldus Information Technology BVWageningenNetherlands

**Keywords:** Parkinson disease, Freezing of Gait, context-aware system, indoor localization, person orientation

## Abstract

**Background:**

Freezing of gait (FoG) is one of the most disturbing and least understood symptoms in Parkinson disease (PD). Although the majority of existing assistive systems assume accurate detections of FoG episodes, the detection itself is still an open problem. The specificity of FoG is its dependency on the context of a patient, such as the current location or activity. Knowing the patient's context might improve FoG detection. One of the main technical challenges that needs to be solved in order to start using contextual information for FoG detection is accurate estimation of the patient's position and orientation toward key elements of his or her indoor environment.

**Objective:**

The objectives of this paper are to (1) present the concept of the monitoring system, based on wearable and ambient sensors, which is designed to detect FoG using the spatial context of the user, (2) establish a set of requirements for the application of position and orientation tracking in FoG detection, (3) evaluate the accuracy of the position estimation for the tracking system, and (4) evaluate two different methods for human orientation estimation.

**Methods:**

We developed a prototype system to localize humans and track their orientation, as an important prerequisite for a context-based FoG monitoring system. To setup the system for experiments with real PD patients, the accuracy of the position and orientation tracking was assessed under laboratory conditions in 12 participants. To collect the data, the participants were asked to wear a smartphone, with and without known orientation around the waist, while walking over a predefined path in the marked area captured by two Kinect cameras with non-overlapping fields of view.

**Results:**

We used the root mean square error (RMSE) as the main performance measure. The vision based position tracking algorithm achieved RMSE = 0.16 m in position estimation for upright standing people. The experimental results for the proposed human orientation estimation methods demonstrated the adaptivity and robustness to changes in the smartphone attachment position, when the fusion of both vision and inertial information was used.

**Conclusions:**

The system achieves satisfactory accuracy on indoor position tracking for the use in the FoG detection application with spatial context. The combination of inertial and vision information has the potential for correct patient heading estimation even when the inertial wearable sensor device is put into an a priori unknown position.

## Introduction

### Background

Freezing of Gait (FoG) is a temporary, involuntary inability to initiate or continue movement lasting just a few seconds, or on some occasions, several minutes [1]. FoG is experienced by approximately 50% of patients with advanced Parkinson disease (PD) [2]. It is described by the patients as a feeling of having the feet glued to the ground and being temporarily unable to start walking again. FoG usually depends on the walking situation. It often occurs at turns, start of walking, upon reaching the destination, and in open spaces [3]. It can also occur when people approach narrow spaces, such as doors, and when people are in crowded places [4]. In a home environment, freezing episodes are usually reported by patients to occur at the same location every day.

In cognitive psychology, attention set-shifting is defined as the ability to move back and forth between tasks, operations, or mental sets in response to the changing internal goals or the changes in the environment perceived through senses. According to Naismith et al [5], the ability to keep different tasks, motor and cognitive, active at the same time is reduced in persons with FoG. The flexibility to shift from one response set to another is therefore impaired which may trigger episodes of FoG. Additionally, this behavior may be emphasized by other provoking features such as increased cognitive load, dual-tasking [6], stress, anxiety, and depression [7]. Irrespective of what causes it, FoG is mostly characterized by a decrease in stride length, an increase in stepping frequency preceding the episode, and the presence of a highly abnormal frequency of leg movements during the episodes [8].

The usual pharmacological way of treating FoG is the same as the general treatment of PD. Research has shown that dopamine treatment helps in reducing the number of occurrences of the symptom, but that it cannot eliminate the symptom completely [9]. Some of the patients developed different ways to deal with FoG on their own. These involved various techniques for solving start hesitation, such as lateral swaying, stepping over someone’s foot, and stepping over lines on the floor. Observations of these techniques led to the development of a theory of sensory cueing as a feasible therapeutic option. Evidence was found that external sensor “cues” (visual, auditory, or haptic) may compensate for the defective internal “cueing system” for initiating and maintaining movement [10]. The research of cueing techniques led to the development of several commercial products intended to help FoG sufferers to improve their gait performance (eg, PDGlasses [11]). The disadvantage of existing commercial products is that they are not adaptable to the walking rhythm of each patient, so that the offered permanent stimulation is not optimally effective [12].

### Wearable Systems and Gait Monitoring

Active monitoring technology has the potential to alleviate FoG through timely episode detection and sensory stimulation. Timely detection is based on online data acquisition of motor symptoms of PD. The usual approach is to use wearable inertial sensors in order to obtain kinematic parameters of the movements of body segments. As already mentioned, gait alterations like short shuffling steps and festinations are characteristic of FoG. Therefore, the analysis of gait parameters is a good indicator of the patient’s state. The foundation of the work on adaptive systems for ambulatory monitoring of FoG was established with the offline detection algorithm based on frequency analysis of leg movements proposed by Moore et al [13]. The authors used accelerometer located at the ankle to take measurements, and associated a frequency band between 3 and 8 Hz with the leg movements when a patient suffered from FoG. Moore's algorithm for FoG was later successfully applied in an online wearable gait assistant for PD patients developed at ETH Zurich [14]. This system used a three-axial accelerometer fixed on the shank and one unique threshold for all patients. Sensor position on the thigh above the knee and at the hip was also tried.

So far, there has been no consensus on the best inertial sensor combination/position for the ambulatory analysis of human gait. The system for FoG detection and gait unfreezing presented by Jovanov et al [15] used an inertial platform consisting of a three-axial accelerometer and two-axial gyroscope placed on the knee. Zabaleta et al [16] performed a pilot study with two PD patients, using sensors to measure acceleration in three axes and angular velocity in two axes. In this study, sensors were used to monitor foot, shank, and thigh movements. The majority of existing systems demonstrate that the best results in FoG detection can be expected when using inertial sensors placed directly at the lower extremities. However, one big drawback of this approach is that strapping sensors on the legs is not optimal for use in daily life.

In the area of assistive technology, user acceptance of technological solutions is crucial. It has been proposed that using one light inertial measurement unit (IMU) fixed on the lateral side of the waist of the user is the most user-friendly position, which also gives satisfying results in gait analysis [17]. The proposal for the sensor placement on the hip can be further supported by the recent results presented by Mazilu et al [18]. Using one sensor fixed at the hip and training a machine learning algorithm specifically for a patient with that patient's data, they achieved excellent results for specificity and sensitivity (both above 98%). However, the presented approach is patient-specific and based on data from a controlled environment. At the moment, the best reported detection accuracy with the sensor placed on the waist and the algorithm targeted at the general PD population is still around 70% to 80% in terms of both specificity and sensitivity.

The amount of information that can be extracted from one sensor device is finite, and it is reasonable to expect that the overall detection accuracy of such a system cannot be higher than the accuracy of a system composed of multiple sensors. Furthermore, wearable inertial sensors currently used for gait analysis are able to sense only physical context of the user, while it is known that the FoG episode onset can also be under direct influence of other types of context (situation, location, and/or cognitive load). It would be nice to keep the ease-of-use of the gait monitoring system composed of only one wearable sensor, and at the same time to enhance its reliability. One way to achieve this improvement is through the use of spatial context, which is the term used to describe a combination of the patient's location and his relation toward elements triggering FoG in his environment.

### Spatial Context in Freezing of Gait

In their home environment, PD patients are likely to encounter narrow passages such as doorways or dynamically changing spaces created by the presence of other people and movable objects such as chairs. When PD patients perceive the space as too narrow for the dimensions of their body, adaptive postural changes during locomotion may be needed to achieve collision-free passage [19]. Experiments with PD patients show that there might be a direct correlation between the width of the narrow space and the tendency for a FoG episode [20]. It has been reported that freeze-like events were successfully provoked near a doorway and that their prevalence significantly increases with the narrowness of the doorway [21]. Furthermore, the measurements of gait based on objective criteria showed that a decreasing door width caused progression velocity to drop approximately 20% in the region preceding the doorway, or immediately after it.

When inferring spatial context in FoG, we are primarily interested in the locomotion behavior of the patients. Examples from literature show that a two-dimensional (2D) point representation on a floor map is sufficient for this kind of task [10]. Contextual triggers of FoG that can be identified from 2D motion in a robust and efficient way under realistic conditions are starting of walking, approaching the destination, approaching narrow spaces, and being near locations where FoG occurs every day. Additionally, many affected people are experiencing FoG during turns. Wider turns seem to be easier for them to perform than axial turns on the spot, and slow turns are easier to perform than rapid turns [22]. A rapid turn on the spot is hard to track using only a 2D point as the representation of the tracking target. The observation of *on-the-spot* turns can be achieved only by precisely estimating angular velocity of the person, which requires additional tracking of the heading angle. This is the reason why we propose the 2D pose, expressed by two floor position coordinates (*x, y*) and one heading angle (*θ*), as the minimal tracking representation to be used in the spatial context inference process for FoG detection.

### Position and Orientation Tracking for Freezing of Gait Detection

One of the main objectives of our research is to discover if spatial context and the principle of direct geometric correlation can effectively be used to improve automatic detection of FoG in a home environment. This objective requires design and development of a technical system that is able to observe people and their environment, along with the ability to apply correct contextual rules using the observed data. The hypothesis of the direct correlation between geometry of the surrounding environment and FoG episodes has so far been tested only in controlled laboratory environments. There is a need for behavioral data of FoG patients from homes, because a clinical environment is not perceived by people in the same way as their natural environment. As a result, freezing episodes do not happen in the artificial setting in the same way that they would happen at home. The lack of domestic behavioral and environmental data of FoG patients is a significant obstacle that must be taken into account during the analysis of requirements for the design of a future context-aware system.

We divided the development process of the system into two principal stages. The goal of the first stage is to establish a people-tracking system for the collection of behavioral data in the homes of people with FoG. Collected data will be used to build the needed contextual model of FoG. In the second stage, the contextual inference part will be added to the existing tracking system with the goal of testing the finalized system through long term deployment. During the first stage, short term, one day long experimental sessions are expected in both clinical settings and home environments. Because of this, the position and orientation tracking system being developed needs to have the properties of a portable system, allowing for fast installation and setup. Besides reliability and accuracy in tracking people's position and orientation, the system also needs to be modular allowing for scalability in the coverage of an indoor space. Additional requirements for permanent deployment are the ability to identify the FoG patient among members of a household, usability on a daily basis, and ultimately, affordability.

Taking into account the above requirements, we have designed a solution for an improved, pervasive context-aware home-based system for PD patients based on distributed sensing. In the development process, we have come to the end of the first stage, where we have obtained a prototype of the indoor position and orientation tracking system. The prototype consists of a network of Microsoft Kinect [23] cameras and one smartphone worn by the patient, and it needs to be tested for accuracy before starting behavioral data collection in the home environment.

The main objective of this paper is to present a functional and architectural solution for the ubiquitous context aware system for FoG detection, with special attention given to the accuracy evaluation of the developed prototype system for indoor position and orientation tracking.

## Methods

### Ubiquitous Monitoring System for Freezing of Gait

**Figure 1:**
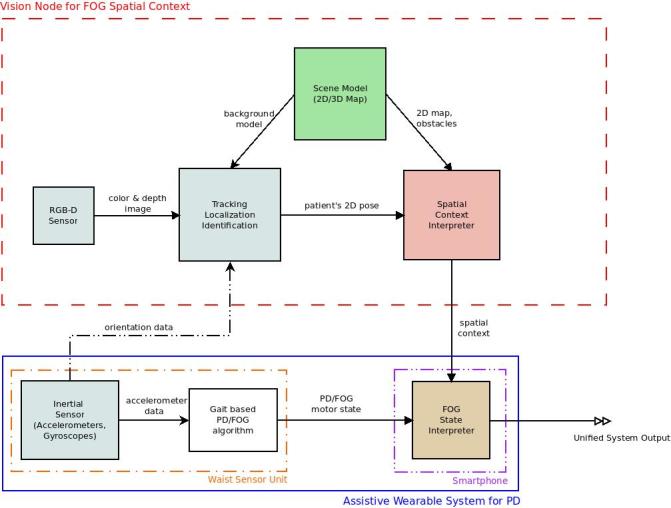
Block diagram for the concept of the ubiquitous monitoring system. The wearable system independently detects FoG based on inertial data (blue rectangle). Gait-based detection is complemented by the user's spatial context from the vision sensor system (red rectangle) in the areas of the home where such a system is present.

In the concept of a ubiquitous FoG monitoring system [24,25], a wearable assistive system is used to monitor gait with inertial sensors and to treat the FoG patient via a cueing device at any time or place during the day. REMPARK (Personal Health Device for the Remote and Autonomous Management of Parkinson Disease) is an example of one such system [26]. The sensing capacity and detection capabilities of the wearable assistive system are expanded with a network of vision sensors installed in the patient’s home environment, placed in the least sensitive areas concerning privacy, such as the living room, kitchen, and hall. The vision system runs image-based tracking, environment mapping, and context inference on a dedicated home gateway server. The concept and the components of the system are presented in the block diagram in Figure 1.

### Distributed Home Vision System

Video cameras and video processing are often used in smart environments for event detection and context inference. Cameras enable the observation of changes in the environment, and at the same time, they are able to provide sub-meter accuracy of indoor localization. Limitations of the usual color (RGB) camera system are its sensitivity to changing lighting conditions, shadows, and occlusions. Active range cameras, such as the Kinect's depth sensor can be applied to overcome the drawbacks of color cameras. Furthermore, one depth sensor is enough to retrieve the three-dimensional (3D) information about the environment compared to a setup of multiple calibrated color cameras usually required for the same task.

**Figure 2:**
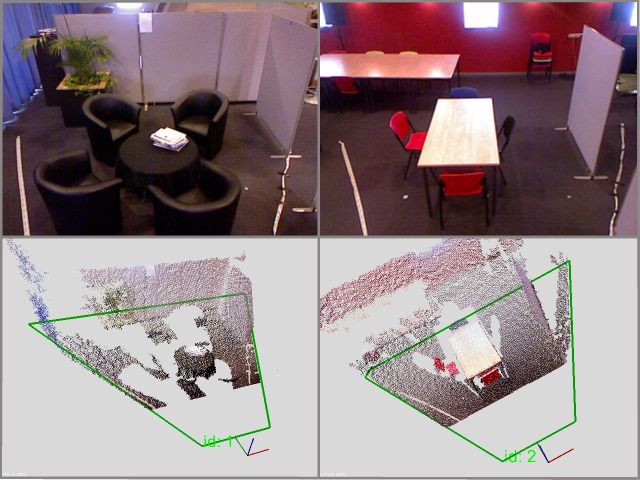
Example of a test bed with two scenes being independently covered by Kinect sensors. Mock-up of a living room on the left and a dining room on the right. Images in the top row depict the point-of-view of the cameras when they are mounted in the overhead position. The bottom row displays colored point clouds of scenes that are obtained from depth sensing. Green trapezoid indicates the area in which it is possible to track people.

To achieve the maximum spatial coverage for each Kinect sensor in an indoor environment with normal ceiling height, we decided to use these sensors in an over-head mounting position. Also, to achieve the most effective coverage inside a home with a minimum number of vision sensors, we decided to use non-overlapping scene coverage with only one or two Kinect sensors per room. An example of the intended spatial coverage is given in Figure 2.

Multiple person tracking and identification should be included in the system since the majority of PD patients live with at least one other person (see Multimedia Appendix 1). Each color and depth (RGB-D) camera in the system is intended to work as an independent vision node in terms of positional tracking. This means that each camera should have the map of the scene in front of it and that it should track people in the coordinates of its own frame. Context rules will be evaluated only for the camera that is tracking the patient. The intended application in unpredictable home environments brings a series of requirements. Besides support for multiple people tracking and patient identification, the vision system should have near real-time performance with at least 15 Hz frequency rate, robustness to dynamic backgrounds, and lighting changes and sub-meter position tracking accuracy.

### Context Inference Process and Freezing of Gait Detection

**Figure 3:**
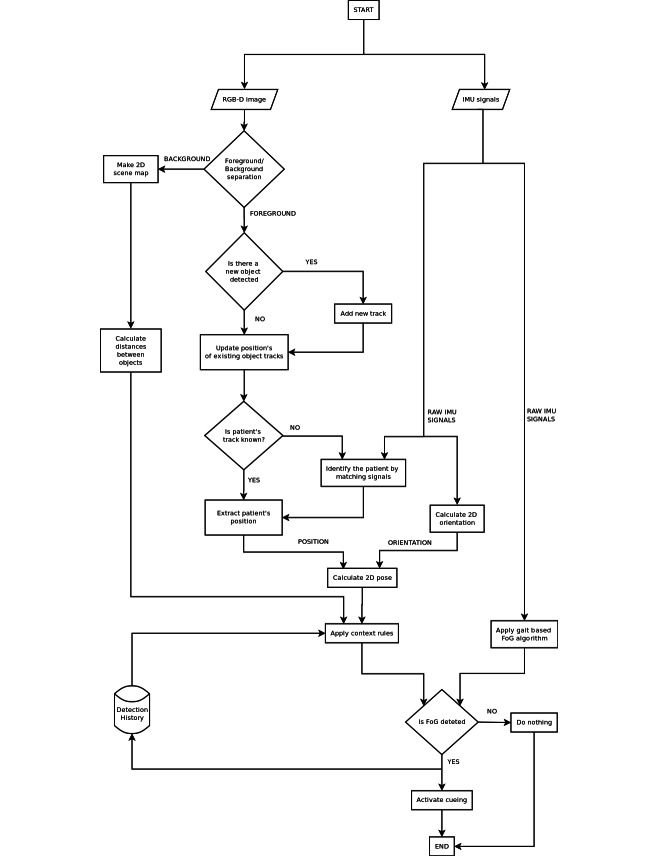
Workflow diagram for FoG detection using the distributed sensor system.

The workflow diagram of the system is given in Figure 3. The diagram shows how one RGB-D camera is paired with a wearable sensor in order to achieve improved FoG detection and cueing actuation. This process can be executed for each RGB-D camera.

Independent elements of the process include 2D position tracking and 2D scene map calculation using RGB-D image, 3D orientation calculation using inertial data from the wearable sensor, and gait-based detection of FoG from inertial signals. These elements have to work independently, so that FoG detection can be achieved using the wearable sensor even when the patient is not in front of the camera.

The main prerequisite for position tracking is background subtraction in each frame. Background subtraction is based solely on the depth image. The background model for subtraction is set by periodic updates of the 3D point cloud of the whole observed scene. These periodic updates are done every few minutes on occasions when no tracked objects are present in the field of view. Furthermore, this background model is used to build the 2D map of the scene, which is used as one of the inputs for spatial context inference.

The foreground image obtained after background subtraction is used to build point clouds for updating the positions of the persons being tracked and to detect any new person in front of the camera. After the detection of new persons, positions of all tracked persons are updated. We are only interested in the position of the patient. If the track of the patient is not identified, the process of matching all known track histories against inertial sensor data is executed. If the match is successful and the patient's track is known, the position of the matched track is used in the calculation of the patient's pose. If none of the tracks in front of the camera are identified as the patient, the camera data is excluded from FoG detection.

Pose calculation involves a combination of the position obtained from the vision tracker and the 2D heading obtained from the wearable sensor. The estimated 2D pose is combined with the 2D map information and history of FoG detections to infer contextual probability of a FoG episode. This probability is published over a wireless network and read by the FoG State Interpreter (FSI) module running on a smartphone device. The FSI module conducts a high level probabilistic fusion of spatial context and gait detector outputs and produces the final system output which can be used to activate cueing.

### System Prototype

The hardware prototype of our distributed sensing system consists of two static Kinect devices and one Samsung Galaxy Nexus smartphone, worn by the user. Each Kinect is connected to its own notebook computer, which acts as a processing unit for data acquisition and also runs one instance of the vision tracking algorithm. The notebooks are connected in a dedicated local area network (LAN) and they are synchronized with respect to time. Each Kinect acquires a depth and color image of resolution 640×480 pixels at a frequency of 30 Hz. The smartphone has connectivity with the dedicated wired LAN over a software access point running on one of the notebook computers. The smartphone reads data from its internal inertial sensors, three-axial accelerometers, gyroscopes, and magnetometers with the frequency of 100 Hz.

After the investigation of available middleware systems for intelligent environments, we chose an open source, community-supported middleware from the robotics domain to develop our distributed sensor system. The Robot Operating System (ROS) [27] is a meta-operating system that runs on top of the real operating system (Linux Ubuntu). A great advantage of using ROS in our project is that ROS program packages provide automatic hardware support and integrated access to various open source processing libraries. The main libraries we use are the Open Source Computer Vision Library [28] and the Point Cloud Library [29]. The algorithms for the vision tracking system were developed in C/C++. The Galaxy Nexus smartphone with Android OS runs the ROS node application, which shares raw inertial data with other nodes in the ROS framework. Image compression and data recording tool packages provided by ROS enable the efficient and synchronized recording of the entire raw sensor data produced in the distributed system.

### Position Estimation

Although the nominal operation range of the Kinect depth sensor is 0.8-3.5 m, our goal was to apply the sensor in the extended range up to 6 m, which more than doubles the area coverage. At the distances greater than 3 m, the quality of Kinect depth sensor data degrades due to noise and low resolution of measurements [30]. The requirement of having a uniform tracking method, which is equally applicable at all distances from the camera, motivated our use of a probabilistic approach to tracking.

Plan-view tracking is a computer vision approach that uses 3D data as input and combines geometric analysis, appearance models, and probabilistic methods to track people on the 2D floor plane [31]. The main prerequisite for successful implementation of any plan-view tracking algorithm is to define the 3D pose of the camera in relation to the floor plane. For our application, we developed a semi-automatic procedure to detect the floor plane with minimal input from the user during the setup of the system. After calculating the floor plane equation in the camera image frame, we define the coordinate frame for 2D position tracking which is set at the base of the camera, on the ground floor level. The known transformation between the camera image frame and its referent 2D tracking frame is used to project a colored point cloud of the foreground onto the floor plane. The projected points are used to calculate three plan-view feature maps (height, occupancy, and color), which are used as observations in the tracking algorithm. Our adaptation of the plan-view tracking algorithm for the Kinect sensor is based on the work of Muñoz-Salinas [32]. This tracking approach uses multiple particle filters, one for each person. A first order linear dynamic model with Gaussian noise is used in the filter's prediction step. The correction step uses a Gaussian mixture of the three feature maps as the observation support.

For FoG detection, it is sufficient to track people when they stand. This can be a mitigating circumstance under real world conditions. When detecting people who are standing, it is sufficient for the system to observe only the 3D environment above a certain height. Setting a height cut-off threshold at around 1.0 m solves two frequent problems in indoor tracking, which are static object occlusions by furniture like chairs and tables, and background updates. Using such a threshold implies that changes in the scene below the height of the threshold do not have any influence, which results in a more robust tracking algorithm.

### Person Orientation Estimation

The combination of the accelerometer, gyroscope, and magnetometer signals from the smartphone allows the estimation of the absolute 3D orientation of the device toward the fixed global coordinate system defined by directions of gravity and magnetic North. The focus of our work in people orientation estimation is not on the development of new fusion algorithms for inertial devices, but it is on the development of methods for the use of existing inertial fusion orientation algorithms in the context of our distributed system.

There are two reasons why the measured orientation of the device cannot be used without adaptation in our tracking system. First, the estimation of the user's (patient's) orientation is needed in the distributed system only when the user is viewed by any of RGB-D cameras. Each camera in the system has its own coordinate system. Therefore, the orientation of the user at the given moment needs to be expressed as the angle in the coordinate system of the camera which performs the tracking, instead of global magnetic-North-referenced world frame. Second, we must strictly differentiate between the orientation of the inertial device and the orientation of the user, and emphasize that they cannot be considered equal. When the inertial device estimating orientation in reference to the global frame is fixed on the body of the user, its orientation in reference to the user's body must be exactly known in order to be able to correctly calculate the user's orientation toward the global frame of reference. In the real-world, every-day scenario, there are no means to exactly know the device orientation in reference to the user, even if the sensor is fixed in the correct position. When the smartphone is placed in a horizontal belt case by the user, there is an uncertainty because the device is not fixed directly on the user. The belt case could actually be positioned anywhere on the belt around the waist.

We have developed two methods for transforming the orientation of the inertial device into the 2D heading of the user, expressed in the referent camera coordinate system. In our methods, we use the very good and proven device orientation estimation algorithm introduced by Madgwick [33]. The algorithm uses numerical integration of the orientation data in the quaternion representation. There are two versions of the algorithm depending on the number and the type of sensors available in the inertial sensor system where it is applied. The basic version of the algorithm is suitable for IMU devices consisting only of gyroscopes and accelerometers, enabling the tracking of rotational and translational movement. This basic version of the algorithm uses gradient descent optimization, which makes it possible to obtain the relative orientation of the device toward the gravity field based on accelerometer input. When referring to this version of the algorithm in the rest of the paper, we will use the name Gravity Relative Orientation Estimation (GROE) algorithm. This basic algorithm is not able to give absolute 3D orientation, since there is no absolute reference in the plane perpendicular to the gravity vector. To achieve complete measurement of 3D orientation in the gravity field, Earth's magnetic North reference system, it is necessary to have the ability to sense the Earth's magnetic field. A MARG (Magnetic, Angular rate, and Gravity) sensor is an extension of IMU, which also incorporates a tri-axis magnetometer. An extended version of the algorithm that can be applied on MARG sensory platform computes its result by numerically integrating changes of orientation measured by gyroscopes, and then correcting gyroscopic measurement errors using a compensation component obtained from the combination of accelerometer and magnetometer measurements. The gradient descent algorithm that uses the combination of accelerometer and magnetometer data takes care of achieving absolute 3D orientation in several iterations after the algorithm initialization. We will refer to this version of the algorithm as the Absolute Orientation Estimation (AOE) algorithm. Both versions of the algorithm are stable, computationally inexpensive, and effective at low sampling rates.

The first method we developed for person orientation estimation uses data only from wearable inertial sensor. The method employs *AOE* algorithm to obtain absolute 3D orientation of the device and relies on the following three assumptions: (1) the sensor device is worn in the predetermined orientation and at the predetermined position relative to the body of the user, (2) the heading is estimated only when the user is standing, and (3) the angle between the magnetic North frame and ground camera frame is known in advance.

**Figure 4:**
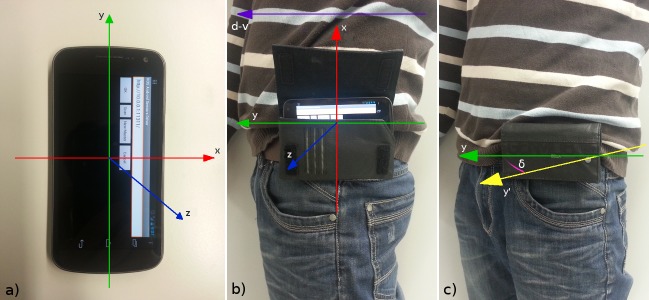
Frame definitions. a) Smartphone reference axes. b) Smartphone in the correct predetermined orientation at the expected position and orientation on the waist. c) Smartphone in the non-expected position and orientation on the waist. There is an angle of error in the transverse body plane between the device's real (green arrow) and expected (yellow arrow) orientation.

We defined the user's orientation as a vector along his dorsoventral axis with the direction from the dorsal to the ventral side of the body. As the predetermined position for placing the smartphone, we chose the left hip. As the reference coordinate system orientation for the smartphone, we set the x-axis facing upward along the anteroposterior axis of the body, the y-axis parallel to dorsoventral axis, and the z-axis facing left from the body along the left-right axis. Expected smartphone positioning is depicted in Figure 4b.

**Figure 5:**
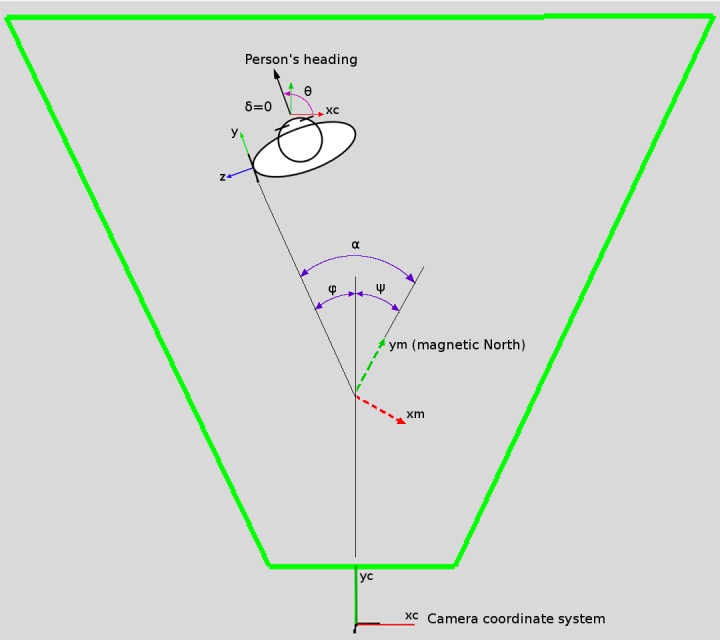
Overhead view of the relations between the different frames in the system.

When the smartphone is in the expected ideal position and orientation on the user's body, the vector of gravity will be along its negative x-axis, while y-axis and z-axis define the plane parallel with the floor (see Figure 4b). Thus, we can obtain the 2D heading of the device in the floor plane by measuring the angle between the y-axis of the smartphone and the axis of magnetic North (*α*) with *AOE* algorithm. Since there is no difference between the presumed direction of the y-axis of the smartphone and the user's heading vector (*δ* = 0), angle α also gives the heading of the user in reference to the magnetic North, as shown in Figure 5. Our final goal is to obtain the heading of the user in the camera frame (*θ*). Two corrections with known static angle values are necessary. To get the user's heading *θ*, first the measurement of the smartphone (*α*) is corrected for angle (*ψ*) between the yc-axis of the camera coordinate system and the ym-axis of pointing to magnetic North. This gives angle *φ*, which defines the user's heading in reference to the yc-axis of the camera coordinate frame. Since user's heading *θ* is always expressed as the angle toward xc-axis, a final correction is executed by adding 90° to angle *φ*.

Our second person orientation estimation method uses wearable inertial sensor data in combination with the classification of the person's orientation conducted in the vision tracking system. The goal of the method is to eliminate the set of assumptions used in the first method, making it more robust and applicable for use in uncontrolled home environments. The method uses the previously-introduced *GROE* algorithm, which estimates the 3D orientation of the device relative only to gravity. As the algorithm can align just two of the inertial device's axes with the plane perpendicular to the gravity (presumed floor plane), this leaves the final angle of the device unknown. To calculate the device's heading in the floor plane, an external reference angle is needed. If, instead of the gravity-magnetic North, we use as the referent frame for the external reference angle the frame in which the camera is currently tracking the user's position, we can eliminate the need for finding the angle between the camera tracking frame and the gravity-magnetic North frame. Furthermore, the assumption of having the wearable sensor in the predetermined position can be eliminated if the external heading reference angle given to the inertial sensor contains information about the true heading of the user expressed in the common frame of reference. Providing the necessary external heading reference is therefore the task of the vision tracking system, because of its ability to observe the user directly in the camera reference system.

**Figure 6:**
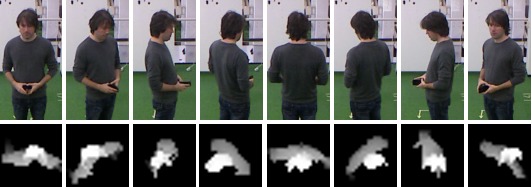
The top row shows eight headings for one person at the same position in reference to the camera. The bottom row contains examples of related height templates used in orientation classification with neural network.

The implemented vision-based orientation classifier was inspired by the work of Harville [34], where the person's plan-view height templates are used to classify eight different headings in the range between 0° and 360° with a 45° resolution for humans standing upright (see Figure 6). Our neural network classification algorithm was trained with the features of 4 persons of different heights. To achieve uniformity of the visual orientation detection in the whole area covered by one camera, training data was collected from people standing at different distances and positions in relation to the camera. The positions for data collection were set using a grid of 0.5×0.5 m rectangles on the floor. People were asked to move horizontally, vertically, and diagonally on the grid, akin to pieces in chess, and to stop in the middle of each rectangle of the grid for one second. During post-processing, a total of 6022 height templates for 4 persons were extracted and labeled with their pertaining classes. The feature vector for classification consists of 443 attributes, the first 441 being normalized pixel values coming from the 21×21 pixel height image template, and the last two being height normalization constant and the number of non-zero elements in the template image. The neural network has an input layer with 443 neurons, a hidden layer with 25 neurons and an output layer with 8 neurons. Classic back-propagation training algorithm with symmetric sigmoid activation function was utilized.

The classification accuracy test on 100 height templates gave 92% correct classifications. During testing under real-world circumstances (ie, when movement paths and poses of people were not in the strict consensus with the eight trained orientations), a significantly higher amount of incorrect classifications was observed. Errors were noticed in classification between opposite directions and also in classification of body poses that differ too much from upright standing. This is the source of the possible error in the heading reference.

When the classifier proposes the orientation reference for wearable system, its accuracy needs to be ensured. A high confidence level for the heading reference can be achieved with the use of two additional sources of information: the quality score of the classification result, and the position history of the person. The quality score of the classification result is calculated using values at output neurons. An eight class neural network has eight output neurons, and the rule is that the output class of the whole classifier is assigned to the neuron with the maximum probability. The output neuron with the maximum probability has a high value when the user's height template is similar to a training template. This probability number can be used as the quality indicator for classification. A high confidence level using the classification quality score is achieved through a temporal process, where the classifier output is tracked for consistency to be above a certain threshold during several consecutive frames. When this consistency holds, the orientation angle represented by the class can be taken as the person's heading proposition. We call this angle *static heading*. To further strengthen the heading proposition and minimize the probability of assigning the opposite direction, the kinematic properties of the person's track are used. Using position history, the velocity vector for the tracked 2D point is calculated. This vector in relation to xc-axis of the referent coordinate frame gives the angle called *dynamic heading*. Ultimately, when the angular difference between the static and dynamic heading is inside a specified error boundary (ie, +/- 15°) for 3 consecutive image frames, the *static heading* is confirmed to be the external heading reference for inertial system.

**Figure 7:**
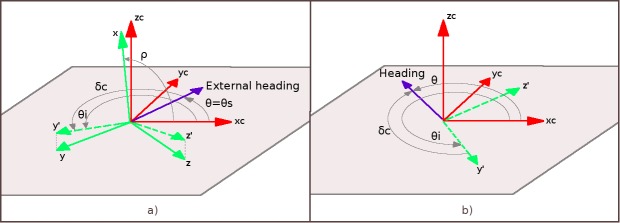
Coordinate frames in the process of fusion of vision and inertial information for orientation estimation. a) The moment in time when the external heading reference is available. b) Using the calculated correction angle to get person's heading at times when only the inertial orientation estimation is available.

When the person is upright and wears the smartphone in the belt case, one of the axes of the device points approximately along the gravity vector, while the other two axes span the plane, which is almost parallel with the floor. This can be seen in Figure 4b, where the x-axis of the smartphone is pointing upward and axes y and z are forming the specified “almost parallel” plane. Since the *GROE* algorithm estimates the angle of orientation of the smartphone toward the gravity, it measures how much the plane formed by y and z axes is deviating from being fully parallel with the floor plane. This angle can be used to calculate the projection of y and z axes on the floor plane. Axes y' and z' shown in Figure 7a are the result of such projection.

The external heading reference angle *θs* is not always available, but only when the vision tracker has a heading proposition of sufficient quality. When the external heading reference angle *θs* is known, it is possible to calculate the value of the correction angle *δc* between the external heading reference vector and the referent orientation axis of the inertial sensor system. In Figures 4b and 4c, the y-axis is set closer to the user's dorsoventral axis, so we choose its projection y' to be the referent orientation axis for the fusion. Figure 7a shows the relation between the x-y-z coordinate frame of the smartphone, the xc*-*yc*-z*c coordinate frame of the camera, and the linking zc*-*y'*-*z' frame used for the fusion at the moment in time when the static heading is known. Correction angle *δc* is calculated as the difference between the angle *θi* of y'-axis and the external heading reference angle *θs*, which at that moment also represents the person's true orientation *θ*.

In the subsequent frames when no external heading reference is available and there is the dependency only on the inertial system orientation estimation, angle *δc* is subtracted from the observed angle *θi* to get the person's true heading *θ*. This is demonstrated in Figure 7b.

### Experiment

The purpose of the experiment was to confirm the functionality of the position and orientation tracking system for different users, and to collect sufficient data for the statistical analysis of the system accuracy. Additionally, we wanted to show that the user's position can constantly be estimated within certain statistical error limits irrespective of his distance from the camera and his orientation. We chose the approach with the known static ground truths for position and orientation to enable an evaluation based on comparing with known referent values. The smartphone position on the waist of the participant was taken as a parameter in this experiment with the objective of assessing how each of the two heading estimation methods adapts to a change in the sensor attachment position.

The experiment had 12 participants (9 male, 3 female), who were recruited from among the staff and graduate students of the Industrial Design Department of Eindhoven University of Technology. The average height of the participants was 174.2 +/- 8.8 cm. None of the participants had gait problems. The area used for walking had dimensions 8×5 m, and it was covered with a green carpet which had a visible grid of squares of size 0.5×0.5 m. Two Kinect devices were set at a height of 2.25 m facing downward with a pitch angle of approximately 25°. The devices were placed to cover the walking area in a non-overlapping manner. A unique world frame for the experiment was set at the corner of the walking area, with its orientation equal to the base frame orientation of Kinect 1. To confirm the uniformity of the magnetic field in the walking area, we executed control measurements of its quality at approximate waist height (1.0 m) before and after the experiment.

**Figure 8:**
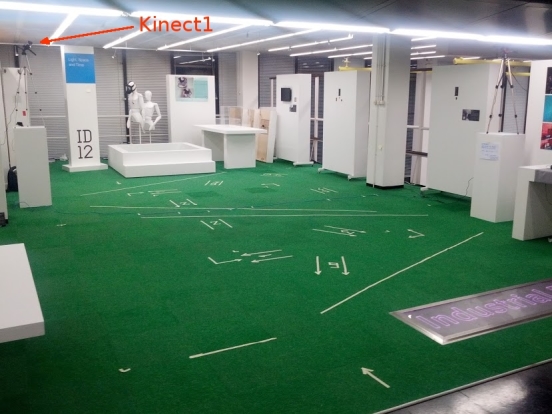
The experiment venue. Markers on the floor indicate the start and end points and numbered reference points for standing in a predefined orientation. Additional markers also show which part of the area is covered by which Kinect device.

On the green carpet surface, markers were placed to indicate points on the floor, where the participants are supposed to stop in predefined orientations (see Figure 8). For each designated pose, two parallel lines of 0.5 m length were drawn on the floor at the mutual distance of 0.25 m. As the reference for measuring the marker position, the center point between two lines was taken.

**Figure 9:**
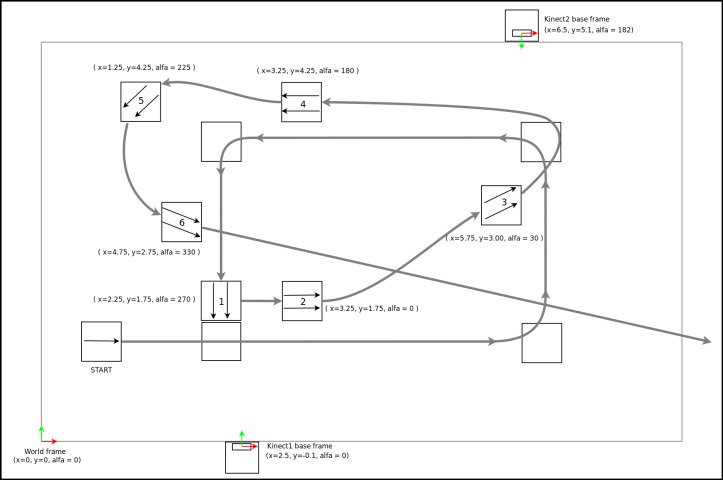
Schematic of marker positions and numbering for walks starting from the left side.

**Figure 10:**
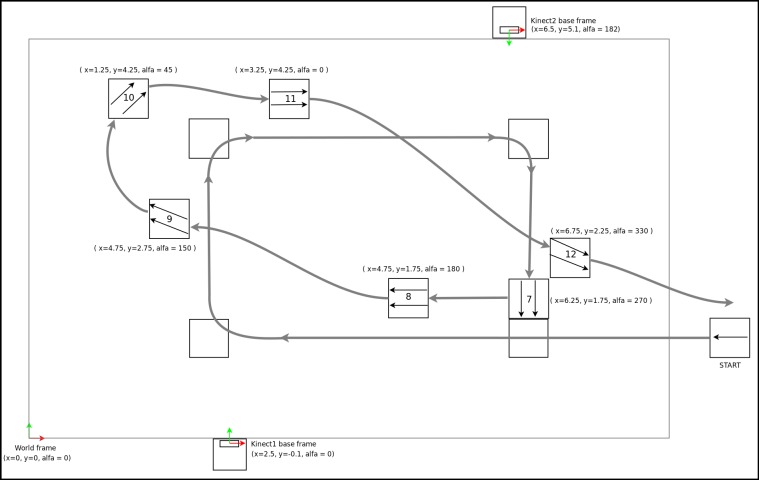
Schematic of marker positions and numbering for walks starting from the right side.

The experimental condition was the sensor attachment position with two possibilities, *Position1* with the smartphone fixed at the iliac crest on the left hip (see Figure 4b) and *Position2* with the smartphone rotated between 50° and 60° around the waist and put on the frontal left side under the belly (see Figure 4c). *Position1* is the expected sensor position for the method using the *AOE* algorithm, while *Position2* is substantially deviating from the expected position for the same method. The second method using the *GROE* algorithm and video orientation classifier has no expected sensor position. The test for each sensor position was split into two walks, one walk with predominantly left turns (see Figure 9) and the other one with predominantly right turns (see Figure 10). Walks were designed with multiple consecutive turns in the same direction in order to induce possible orientation bias. Participants were instructed to walk to each marked position, where they were told to stand still for 3 seconds before continuing toward the next marked point (see Multimedia Appendix 2). The procedure was repeated for each subsequent point. Each test walk lasted around one minute. Each participant first did two walks for condition *Position1*, followed by two walks for condition *Position2*.

During the experiment, color images and depth data of each Kinect were recorded along with the data from the smartphone which encompassed raw accelerations, orientation, magnetometer measurements and calculated orientations for *GROE* and *AOE* algorithms. Estimation of the positions obtained from the video tracking algorithm along with the absolute heading estimation angle for the two orientation estimation methods were stored in a SQL database. Post-processing consisted of annotation of frames when participants were standing still on the marked floor positions and calculation of the average position coordinates and heading angles from sensor data. A video segment of around one second was extracted each time a participant stood still at a reference point.

The vision-based position tracking algorithm gives a new estimation of the position for each frame. With a 30 Hz frame rate, approximately 30 position estimations were available to calculate the average value of the *x* and *y* coordinates during a one second video. Average values with a sufficiently small standard deviation (<0.04) were taken as the measured position coordinates. In total, 288 pairs of position coordinates were obtained (12 participants × 12 reference points × 2 sensor attachment positions). The average value of the heading angle was calculated using temporal alignment of inertial signals with video segments. For the first method using *AOE* approximately 80-100 orientation estimation values were extracted for each 1 second video segment to calculate average angle value. In total, 288 average angle values were calculated. For the second method using *GROE*, the combination of vision-based orientation classification information and smartphone inertial information was collected at the smallest common denominator update rate, which is the rate of the video tracking algorithm. Around 30 orientation estimates were produced each time a person stood on a reference point. The total of 288 average angle values was expected, but orientation was not registered due to an algorithm failure, in 11 out of 288 cases.

## Results

### Position Estimation

Calculated position values from all test walks were aggregated on a per point basis to enable comparison with reference values. Statistical results (see Table 1) include average value, average error, and root-mean-square error (RMSE) for each of the two position coordinates at each stopping point. Under the presumption of normal distribution, the average error value is an indicator of the presence of a bias in the measurement. In our experiments, the overall randomness of the error values does not point to any significant positive or negative bias, or bias in any of the coordinates. The RMSE, which is a good measure of accuracy, indicates that the estimated position was on average in the majority of points 0.16 m or less from the true position.

### Person Orientation Estimation

The results of the estimation of person orientation closest to the ground truth were expected for tests with the sensor in Position1 when all assumptions needed to get the correct result were satisfied. The results for Method1 (*AOE* algorithm) with the smartphone in Position1 are reported in Table 2. The average angle value for a stopping point (each row in Table 2) was calculated from the set of direction angles estimated for each of the 12 participants. The average angle was compared with the point's reference angle value to give the average error and RMSE. We also added the observation of the maximal error, extracting a single case when the participant's orientation was furthest away from the ground truth.

The average error values do not point to the existence of any specific bias. We took the highest observed value of the RMSE as the reference for error. Statistically, an average error of 17° can be expected if the initially assumed conditions about smartphone placement and upright walking posture hold.

Table 3 provides the data for the comparison of the two different smartphone attachment positions when Method1 (*AOE* algorithm) was used. The data in the table was obtained by aggregating on a per participant basis. This means that to get the data of one row in the table statistics were based on a set of 12 different orientations calculated for the stops of one person. The most notable observation is the uniformly negative angle of the average orientation error obtained for Position2. This negative angle is anticipated considering the orientation change of the smartphone performed for the tests with Position2. The average error values in each row of Table 3 indicate how much the smartphone was rotated around the anteroposterior axis for each participant. Negative angle values of the average error for Position2 closely match values of the RMSE.

Evaluation results of the person orientation using Method2 (see Table 4) are similar to those achieved in Method1 (see Table 2), with the exception of bigger RMSE values maximum errors, which indicate worse behavior of Method2 at certain moments.

Our expectation is that Method2 is able to compensate for the unknown orientation change of the attachment point of the smartphone. The adaptive nature of the method is visible in Table 5 from the fact that there is no significant difference in the observed average errors and RMSE between the two attachment positions.

**Table 1:** Statistical results for position measurements of reference points.

Point ID	Coordinate	Ref. value [m]	Avg. value [m]	Mean error [m]	RMSE [m]
1	x	2.25	2.21	-0.04	0.07
	y	1.75	1.74	-0.01	0.06
2	x	3.25	3.14	-0.11	0.16
	y	1.75	1.66	-0.09	0.13
3	x	5.75	5.65	-0.10	0.15
	y	3.00	3.02	0.02	0.05
4	x	3.25	3.23	-0.02	0.09
	y	4.25	4.19	-0.06	0.10
5	x	1.25	1.22	-0.03	0.06
	y	4.25	4.31	0.06	0.10
6	x	1.75	1.64	-0.11	0.16
	y	2.75	2.77	0.02	0.06
7	x	6.25	6.20	-0.05	0.07
	y	1.75	1.89	0.14	0.20
8	x	4.75	4.79	0.04	0.08
	y	1.75	1.93	0.18	0.25
9	x	1.75	1.73	-0.02	0.07
	y	2.75	2.72	-0.03	0.06
10	x	1.25	1.14	-0.11	0.16
	y	4.25	4.21	-0.04	0.08
11	x	3.25	3.17	-0.08	0.13
	y	4.25	4.19	-0.06	0.10
12	x	6.75	6.71	-0.04	0.08
	y	2.25	2.27	0.02	0.06

**Table 2:** Statistical results aggregated per marker point for person orientation estimation method using *AOE* algorithm (Method1) with the smartphone on the hip (Position1).

Point ID	Ref. angle [°]	Avg. angle [°]	Avg. error [°]	RMES [°]	Max. error [°]
1	270	278	8	11	24
2	0	-2	-2	8	20
3	30	37	7	13	24
4	180	181	1	7	13
5	225	231	6	10	19
6	330	333	3	7	13
7	270	269	-1	10	26
8	180	181	1	9	16
9	150	150	0	9	17
10	45	41	-4	12	22
11	0	-8	-8	13	23
12	330	331	1	12	22

**Table 3:** Statistical results aggregated per participant for the orientation estimation method using *AOE* algorithm (Method1) with two sensor attachment positions.

	Position1		Position2	
Participant	Avg. error [°]	RMSE [°]	Avg. error [°]	RMSE [°]
1	-8	9	-66	66
2	-7	13	-41	42
3	3	8	-60	62
4	3	5	-60	60
5	3	5	-43	43
6	7	8	-55	55
7	-8	8	-62	63
8	-6	14	-57	57
9	8	8	-50	50
10	11	11	-47	47
11	13	15	-58	58
12	5	7	-39	40

**Table 4:** Statistical results aggregated per marker point for orientation estimation using vision based classification and the *GROE* algorithm (Method2) with the smartphone on the hip (Position1).

Point ID	Ref. angle [°]		Avg. angle [°]	Avg. error[°]	RMSE [°]	Max. error [°]
1	270	276		6	15	47
2	0	2		2	15	44
3	30	50		20	21	32
4	180	188		8	10	15
5	225	236		11	17	37
6	330	334		4	13	33
7	270	272		2	14	27
8	180	187		7	16	35
9	150	143		-7	24	32
10	45	40		-5	17	32
11	0	-6		-6	13	22
12	330	313		-17	18	28

**Table 5:** Statistical results aggregated per participant for the person orientation estimation method using vision based classification and *GROE* algorithm (Method2) with two sensor attachment positions.

	Position1		Position2	
Participant	Avg. error [°]	RMSE [°]	Avg. error [°]	RMSE [°]
1	11	28	5	13
2	-2	19	3	14
3	-4	20	4	21
4	10	14	13	22
5	-3	15	4	14
6	4	17	6	16
7	0	13	1	14
8	4	12	11	17
9	-3	12	0	13
10	0	13	-6	18
11	0	14	13	12
12	8	12	9	16

## Discussion

### Technical Properties

The final goal of the experimental measurements of the position orientation tracking subsystem is to properly model its output as a virtual sensor that senses 2D poses and has known characteristics in terms of accuracy and noise. This will enable the output of the patient localization subsystem to be combined with environment mapping data using probabilistic principles, similar to the ones already developed in robotics [35].

The position estimation errors in Table 1 have two principal sources. The first source is the tracking algorithm based on the noisy depth sensor data. The second source is the random nature in which participants arrived at marked points, since during the experiment they were allowed to stop anywhere along the 0.5 m marker line inside a target square. With the current experimental design, it is impossible to separate the contribution of each source to the obtained position errors, so we will impose a strict rule and assign the whole error to the tracking algorithm.

The RMSE is equal or less to 0.16 m for all the measurement points in Table 1, except for points 7 and 8. A greater error in these points can be explained by the combination of body position, camera placement, and depth sensor characteristics. When a person is sensed by a depth camera, depth measurements are taken only on the side of the body directly exposed to the camera. Close to the camera in the overhead position (points 1 and 2), a depth sensor will collect more 3D points from the head and upper shoulder, which are the parts closer to the vertical body center. At the middle distances (2-4 m) from the camera (points 7 and 8), the depth sensor will collect the majority of points from the exposed side of the body. In point 7 this part of the body is at the back, and in point 8 at the right side of the body. This anomaly happens only when people are exposed to the camera under orientation angles close to 0°, 90°, 180°, 270°, and 360°. When a person is oriented diagonally toward camera, more depth points are taken from the body center. At the bigger distances (after 4 m), depth sensor noise and smaller occupancy values influence the tracking algorithm to give more significance to height values, estimating a position more toward the true center of the person.

The comparison of the average orientation errors for the same points across Tables 2 and 4 implies that there was no significant magnetically-caused bias at any marker position. The accuracy comparison based on the maximum RMSE and maximum errors in the same tables reveals that the first method with *AOE* algorithm performed better when the sensor was in Position1. The RMSE values, and especially the maximum error values, presented in Table 4 indicate that Method2 in its current implementation under-performs in terms of accuracy. The cause for this is incorrect static orientation (45° left or right from true value) registered as the external heading reference at certain moments. This could be improved by decreasing the allowed angle error between static and dynamic headings. However, this decrease in error angle can prolong the time necessary to fulfill conditions for registering the external heading reference after entering the camera scene. With the current setup, the detection time for the external reference of a person's heading can sometimes be delayed for one second, depending on how close the trajectory of the movement is aligned with the eight principal orientations of the classifier. This delay is also the reason for the algorithm failure in 11 of the recorded cases. On the positive side, our adaptive vision-inertial sensor information fusion method performed as predicted in conditions of unknown sensor placement, evidently outperforming the non-adaptive method, as seen in the results for Position2 in Tables 3 and 5.

### Implications for Freezing of Gait Monitoring in a Home Environment

For FoG detection based on location, it is of great importance to achieve sufficient accuracy when measuring the distance between the patient and an obstacle. For the case when the system needs to observe that the patient is passing through a door frame, necessary accuracy of location sensing is in the range of several decimeters. The same is true for the case when the patient is standing next to an object, such as a chair. Proximity to an object in a congested space can easily be inferred when the person is standing at a very short distance (<0.4-0.5 m). To set the criteria for sufficient accuracy, we can use the literature about the minimal distance from objects that was observed for people during locomotion behavior. According to Weidmann [36], a person walking in a corridor keeps on average a minimal distance of 0.25 m to a wall made of concrete and 0.20 m to a wall made of metal. Obstacles in a general environment are avoided with a gap of at least 0.10 m. The achieved result of RMSE=0.16 m is acceptably close to the given minimal distance values.

The heading of the patient should be observed with the goal of inferring if he is facing any specific landmark on the map. When observing the patient's relation with the landmark, such as having the intention of going through a door or facing a kitchen sink, the heading error of 15-20 degrees left or right from the true angle is acceptable, because such an error cannot change the perception about the patient being generally directed toward the object. As the indicator of the orientation accuracy for each method we took the worst RMSE value in its related table (Table 2 for Method1; Table 4 for Method2). For Method1 we obtained RMSE=17°, which is satisfying in relation to the acceptable error of 15-20^o^. Method2 gave RMSE=24°, which falls just outside of the desired error range. Results in Table 5 show similar RMSE for different attachment positions of the sensor (28° vs 22°) which proves that Method2 is able to adapt to an unknown sensor attachment situation.

In conclusion, for the orientation data collection from patients in controlled conditions, the recommendation is to use the smartphone and *AOE* algorithm, because it is the simplest solution with acceptable accuracy. For uncontrolled conditions, like a home environment, we propose to apply the method based on the fusion of vision and inertial sensor information. A successful real-world application of this method depends on the improvement of the algorithm to achieve faster detection of the person's true orientation after entering the camera scene.

### Implications for Freezing of Gait Monitoring in a Clinical Environment

We had a chance to deploy the prototype of the tracking system in its current form in a clinical environment, where we observed a rehabilitation session of one 80-year-old PD patient with a 13-year history of PD and high affinity toward FoG. The purpose of the test was to confirm that the system can be used as a portable system and to find out its applicability and value for clinical rehabilitation. Two Kinects were set on special 2.5 m high tripods and put in the corners of two rooms (both size 4.5×4.5 m) in the rehabilitation facility. The time necessary to setup the system was around 15 minutes. The patient was wearing the smartphone at the hip position. First, the usual therapy protocol which included warm-up, Get-up and Go exercise and walking with the visual and audio cues inside one of the rooms was observed. Our first addition to this protocol was the exercise for the patient which included quarter turns on the marked position in front of the camera. The second addition to the protocol was the exercise in which the patient started by sitting on the chair in one room and then had to walk to the chair in the other room passing two doorways and a hallway in-between the rooms. Each Kinect covered a part of one room with a doorway and a chair.

The quarter turns exercise gave us the opportunity to observe the influence of the patient’s stooped posture on positional tracking and visual orientation classification. We had been aware that the change of the posture might influence the final tracking output, although we are using a non-articulated tracking model. Initial qualitative results indicate that the stooped posture has minor influence on the positional tracking, while its influence on the orientation classification is higher than expected, which was manifested as an increase in the rate of incorrect classifications.

The exercise with sitting and walking between the rooms was a combination of Get-up and Go exercise, door passing, and on-the-spot-turning, and it was very demanding for the observed patient who experienced multiple FoG episodes. During the exercise the system was able to track the patient when he was standing, walking, and sitting. Taking this into account, we envisage the use of this tracking system in the clinical setting. We base the exploitation possibilities of the system on the idea of the quantitative assessment of the effectiveness of the therapeutic tests, in order to monitor the long-term advancement of the patient. Since the system uses 3D data, it can measure the height of the patient and give his temporal height profile. Height is useful during a sit-to-stand test to measure posture transition times. Furthermore, the system can collect positional and velocity data to compare walking with and without applied visual and audio cues, and to objectively measure the effect that cues have on the patient when walking straight. By tracking orientation, the same approach can also be applied to the evaluation of the patient's response to cueing during turns.

### Limitations of the Study and Future Work

There were several limitations to the presented experimental study of the accuracy of this system. The main limitation is that the study was conducted in healthy people, who were able to maintain an upright posture. The usual posture of PD patients is a stooped posture, and the future system should accommodate for that. This is especially important for template based recognition of static orientation. The next iteration of the prototype will try to take this fact into account. The benefit of developing the new specialized classifier is that by being able to detect the stooped posture, the system will have additional information to infer the general PD state of the patient. Moreover, this information could be used to improve the identification of the patient during long-term system deployment. Additional data from real PD patients will be needed in order to perform quantitative, statistical evaluation of the posture change influence on the system accuracy.

Position accuracy was measured only for persons who were not occluded. Position measurement with multiple persons would offer better insight into position errors caused by partial occlusions. Furthermore, positions and orientations were only analyzed in static cases. Analysis of the dynamic properties would offer better insight into the characteristics of the system. For this kind of experiment, it would be necessary to have a tracking system with higher accuracy and adequate spatial coverage. A comparison with the Vicon system [37] could be a possible solution.

In the presented orientation tracking methods, the assumption of upright posture needs to be upheld to obtain accurate results. The final orientation algorithm should be aware of the current posture of the person. This calls for the development of an even more contextually aware system.

### Conclusions

In this work, we presented a solution that rethinks the problem of FoG detection and monitoring from the standpoint of technology that could be offered in the context-aware homes of the future. The most interesting novelty from the medical aspect is that we decided to form technical prerequisites for the collection of patient data about FoG which takes into account external contextual factors regarding the symptom and the relation of the patient to the environment.

We proposed using a combination of two technologies: 3D vision and wearable sensing with smartphones, which have developed a strong commercial presence in recent years. It is expected that this trend will continue in the future with wearable sensing offering smaller and more energy efficient devices, and 3D vision cameras offering better resolutions and smaller frame factors.

The study of the characteristics of the system prototype showed that at the current moment, we have a system that has sufficient position tracking accuracy for use in the intended FoG-monitoring application. The study of orientation algorithms gave us the necessary insight into the properties of smartphones for indoor orientation tracking in the context of FoG. The proposed method of data fusion for orientation tracking showed not only how it can improve usability, but also disclosed the factors that need to be improved. Future work goes in the direction of the improvement of the current system prototype toward home deployment and pilot experiments with PD patients. To enable the deployment of the system in real homes with multiple people, long-term identification based on inertial and vision sensor data matching needs to be implemented. In addition, to collect the data for contextual modeling, preparations are being made to undertake recordings of the daily activities of people with FoG in their homes using the current prototype of the system.

## Multimedia Appendix 1

An example scenario for multiple people tracking and re-identification. One camera covers the living room space, while the other is installed in the dining room. The scenario depicts a caretaker and a patient at home with two visitors. The goal is to sustain identities for all the subjects in spite of short term occlusions, pose changes, and changes between cameras.

## Multimedia Appendix 2

An example of pose tracking during one test walk. Orientation is estimated using the proposed camera and wearable data fusion algorithm. The red arrow shows the estimated orientation of the person. The purple arrow shows how the orientation would be without correction from the vision-based system.

## Abbreviations

2D: two-dimensional
3D: three-dimensional
AOE: Absolute Orientation Estimation
FoG: Freezing of Gait
FSI: FoG State Interpreter
GROE: Gravity Relative Orientation Estimation
IMU: Inertial Measurement Unit
LAN: local area network
MARG: Magnetic Angular Rate and Gravity
PD: Parkinson disease
RGB: red green blue
RGB-D: red green blue and depth
RMSE: root mean square error
ROS: Robot Operating System
SQL: Structured Query Language
